# *Ginkgo biloba* Extract Drives Gut Flora and Microbial Metabolism Variation in a Mouse Model of Alzheimer’s Disease

**DOI:** 10.3390/pharmaceutics15122746

**Published:** 2023-12-08

**Authors:** Ting Yu, Yueyang Xing, Qi Gao, Dandan Wang, Hongzhuan Chen, Hao Wang, Yongfang Zhang

**Affiliations:** 1 School of Integrative Medicine, Shanghai University of Traditional Chinese Medicine, Shanghai 201203, China; 13454113795@163.com (T.Y.); gaoqi@xingling.com.cn (Q.G.); 2 SPH XingLing Sci. & Tech. Pharmaceutical Co., Ltd., Shanghai 201203, China; xingyueyang@xingling.com.cn (Y.X.); wangdandan@xingling.com.cn (D.W.); 3 Department of Clinical Pharmacy, Institute of Interdisciplinary Integrative Medicine Research, Shuguang Hospital, Shanghai University of Traditional Chinese Medicine, Shanghai 201203, China; hongzhuan_chen@hotmail.com; 4 Department of Pharmacology and Chemical Biology, Shanghai Jiao Tong University School of Medicine, Shanghai 200025, China; 5 Shanghai Universities Collaborative Innovation Center for Translational Medicine, Shanghai Jiao Tong University School of Medicine, Shanghai 200025, China

**Keywords:** EGb, gut flora, intestinal metabolites, non-targeted metabolomic, metagenomics

## Abstract

Alzheimer’s disease (AD) is a complex neurodegenerative disease. Numerous investigations have demonstrated that medications that regulate the “brain–gut” axis can ameliorate disease symptoms of AD. Studies have shown that *Ginkgo biloba* extract (EGb) is involved in intestinal metabolism to meet the goal of illness treatment. EGb is currently utilized extensively in the clinical prevention and treatment of cardiovascular and cerebrovascular diseases. However, the regulatory effect of EGb on intestinal flora and its metabolites in AD pathology remains largely speculative. In this study, the Morris water maze test showed a significant improvement of spatial memory in the AD mouse model (APP/PS1 mice) after EGb treatment. We next confirmed the positive effects of EGb on the gut flora and metabolites of APP/PS1 mice and further showed that EGb treatment reshaped the disturbed gut microbiome, in particular by reducing the *Firmicutes/Bacteroides* ratio and increasing the abundance of *Bacteroidetes*, *Uroviricota*, *Streptophyta*, and *Spirochaetes*. Meanwhile, a non-targeted metabolomics analysis showed that EGb treatment significantly reversed the dysfunction of the microbial metabolic phenotype by altering *Limosilactobacillus* and *Parvibacte*, with 300 differential metabolites modulated (131 up-regulated, 169 down-regulated). Our findings highlight the significant regulatory impact of EGb on intestinal microflora and microbial metabolism in AD mice models and provide a potential therapeutic strategy for AD.

## 1. Introduction

Alzheimer’s disease (AD) is the most common neurodegenerative disease in dementia, characterized by synaptic dysfunction and brain atrophy, with toxic amyloid plaques and neurofibrillary tangles [1]. There were over 50 million cumulative AD patients worldwide in 2020, and it is expected that this number will increase to 75 million by 2030 and exceed 130 million by 2050 [2]. According to the World Alzheimer Report 2022, the National Dementia Program is a policy priority [3]. The US Food and Drug Administration (FDA) has currently approved numerous medications for the treatment of AD, including Tacrine [4], Donepezil [5], Galanthamine, Rivastigmine, Memantine [6], Aducanumab and Lecanemab [7]. In addition, functional magnetic resonance imaging (fMRI) and functional near-infrared spectroscopy (fNIRS), as effective tools for measuring neurocognitive function, have been used to clinically detect cerebral blood perfusion in patients with cognitive impairment [8]. However, the treatment for AD can only improve symptoms and cannot fundamentally cure AD patients. The development of drugs that affect disease progression is still in its infancy. Therefore, it is of the utmost importance to develop more effective treatments for AD.

Recently, it has been reported that the human gut flora plays a crucial role in maintaining the normal physiological conditions and health of the human body and that the composition and basic malfunction of the gut flora contribute to the pathophysiology of most chronic diseases [9,10]. It has also been shown to regulate the physiological components of neurodegenerative disorders [11]. There is a multitude of evidence indicating that the gut flora and metabolites may alter the pathogenic features of AD by influencing metabolism, supporting the “brain–gut” axis theory [12,13]. The “brain–gut” axis is based on the bidirectional interaction between the brain and the gut. On the one hand, intestinal microbes connect with intestinal cells, the enteric nervous system, and the central nervous system via metabolic and neuroendocrine pathways, and they can also regulate pathogenic and cognitive disorders of AD. On the other hand, the brain regulates the secretion of signaling molecules from immune cells and neurons, affecting the composition of the gut microbiome [14,15].

*Ginkgo biloba* extract (EGb) is an effective medicinal component extracted from ginkgo biloba, which mainly contains flavonoids, terpenoids, and organic acids. Traditionally, EGb has been used to treat cardiovascular and cerebrovascular diseases with a variety of pharmacological activities [16,17]. Studies have found that EGb can improve non-spatial learning memory and spatial learning memory in AD model mice [18,19]. Clinical studies have shown that EGb has an encouraging effect in improving cognitive impairment and psychiatric status in patients with AD or Vascular Dementia (VaD). In 2018, nine countries, including China, Germany, and Singapore, jointly released the Clinical Reference for EGb in the treatment of dementia mild cognitive impairment, with some evidence supporting the inclusion of EGb as part of AD or VaD treatment [20]. In addition, EGb can reduce the expression of intestinal transporters by regulating intestinal flora composition, which is beneficial for intestinal health, enhances immunity, and enhances intestinal morphology [21,22]. The hypothesis that EGb alters gut bacteria to enhance cognition in AD model mice has not yet been confirmed. Therefore, metagenomics and metabolomics are used in this study to characterize the entire intestinal microbial community (archaea, bacteria, Eukaryota, and viruses) of AD model mice before and following EGb treatment in order to identify the changes in microbial diversity and richness, as well as genes, functions, and metabolism pathways.

## 2. Materials and Methods

### 2.1. Animals and Treatments

Male APP/PS1 transgenic mice (expressing human mutant APP695 carrying the Swedish mutations and human PS1 mutations) and wild-type (WT) C57BL/6J littermates (all 6 months old) were purchased from Nanjing Model Animal Resource Center (Nanjing, China) and randomly allocated to different groups. The animals were kept at a constant room temperature and humidity in a 12 h light/12 h dark cycle with free access to tap water and food. After one week of adaptation, all mice were randomly divided into four treatment groups: (1) vehicle-treated WT mice (WT), which were administered normal saline; (2) EGb-treated WT mice (WT + EGb), which received a daily intragastric administration of 100 mg/kg EGb; (3) vehicle-treated APP/PS1 mice (APP/PS1), which were administered normal saline; and (4) EGb-treated APP/PS1 mice (APP/PS1 + EGb), which received a daily intragastric administration of 100 mg/kg EGb (*n* = 5 per group). EGb was administered via the intragastric route once a day for 2 months.

To avoid contamination, fresh stools were collected from each mouse after 2 months of administration. A total of 20 stool samples were collected, immediately frozen with liquid nitrogen, and then stored at −80℃ for microbiome and metabolome analysis. All animal experiments in this study were reviewed and approved by the Ethics Committee of Shanghai Jiao Tong University School of Medicine (A-2021-019).

The EGb used in this experiment was GBE50, the product of SPH XingLing Sci & Tech. Pharmaceutical Co., Ltd. (Shanghai, China). GBE50 is a standardized EGb equivalent to the German standardized product EGB761, which has been approved by the China Food and Drug Administration (approval number Z20000049). It consists of 44% flavonoids and 6% lactones and has been widely used in clinical practice [23,24].

### 2.2. Morris Water Maze

The Morris water maze test was conducted using a black circular pool with a diameter of 120 cm, a height of 50 cm, and a depth of 25 cm. Water-soluble non-toxic white paint was dissolved in the water at 22 ± 1 °C. The entire water maze experiment was split into two phases: a 5-day learning test and a 1-day probing trial test. The first stage of the learning test involved training each mouse four times independently to locate a hidden platform that was 1.5 cm below the water surface in 60 s over the course of 5 consecutive days. If the mouse located the platform, it was permitted to stay there for 10 s. The mouse was sent to the platform and kept there for 10 s if it did not discover it within 60 s. The platformless probing test was conducted 24 h after the conclusion of the learning test. The underwater platform was taken away, and the mouse was left to swim for 60 s; then, its performance was recorded with an automatic tracking system to assess memory retention.

### 2.3. Metagenomic Sample Preparation and Analysis

Stool samples were frozen at −80 °C before DNA extraction and analysis, and genomic DNA was extracted using a Magnetic Soil and Stool DNA Kit following the kit instructions. All DNA samples were analyzed using agarose gel electrophoresis to determine the purity and integrity of the DNA, and the DNA concentration was analyzed by Quhit. Sequencing libraries were generated using a NEXTFLEX™ Rapid DNA-Seq Kit. The qualified DNA samples were randomly disrupted using a Covaris M220 ultrasound apparatus, and the entire library was prepared after the fragments with a growth degree of about 400 bp were randomly disrupted. After its construction, the library was detected with Agilent 2100. After the correct insert size was obtained, the effective concentration of the library was accurately quantified by PCR (the effective concentration of the library was >3 nM). DNA sequencing libraries were sequenced in-depth on the Illumina Hiseq Platform at Allwegene Company (Beijing, China).

The predicted gene sequences of all the samples were clustered using CD-HIT software (v4.6.1, 5 July 2022), and the longest gene of each class was taken as the representative sequence to construct the non-redundant gene set. SOAPaligner software (soap2.21release, 12 August 2022) was used to compare the sequencing data with the non-redundant gene set in order to collect information on the abundance of the gene in the corresponding sample. The non-redundant gene set was compared with the NR database and classified into different taxonomic groups using the diamond BLAST tool. The relative abundance, the bioinformatics analysis of the abundance cluster heat map, the principal component analysis, and the partial least squares discrimination analysis were based on the genus information from each sample. The gene function was annotated using and searched against the function annotation database KEGG and GO.

### 2.4. Non-Targeted Metabolomic Sample Preparation and Analysis

Next, 50 mg stool samples were placed in tubes for metabolite extraction, and after vortexing, the samples were homogenized at 35 HZ and treated with ultrasound. Then, the supernatant was obtained after centrifugation, and the quality of the supernatant was controlled and analyzed. Metabolites were detected using ultra-high-performance liquid tandem chromatography–quadrupole time-of-flight mass spectrometry (UPLC-QTOFMS). In order to better analyze the data, a series of preprocessing was carried out on the original data, including the simulation of the missing value recoding and data normalization. According to the expression of metabolites in different samples, correlation heat map analysis, PCA, and PLS-DA analysis were performed on the samples to evaluate the similarity of the samples within a group and the differences in the samples between groups. All metabolites identified by mass spectrometry were compared with the KEGG and HMDB databases to obtain the annotation information of metabolites in the databases. Generally, the threshold *p* < 0.05 and variable importance in projection (VIP) > 1 were used to screen differential metabolites.

### 2.5. Association Analysis of Metagenomics and Metabolomics

We selected gut microbial species enriched in the drug treatment group and the control group with *p* < 0.05, and the significantly abundant metabolites were defined as a log2fold change (FC) > 1, *p* < 0.05, and *q* < 0.05, with 30 metabolites included. Spearman’s correlation coefficient was calculated based on features within the metagenomic and metabolomic data. The heat map was stratified and clustered according to the correlation distance to represent the correlation pattern of species metabolism.

### 2.6. Statistical Analysis

The two-sided unpaired Student’s *t*-test and one-way ANOVA, followed by the Holm–Sidak test, were used to compare the difference between two independent samples. The comparisons of the groups were performed by two-way analysis of variance (ANOVA) followed by Bonferroni’s post hoc multiple comparison tests. All data were expressed as means ± SEM, and *p* < 0.05 was considered statistically significant. Graphical presentations were developed with GraphPad Prism (version 8.0) software.

## 3. Results

### 3.1. EGb Ameliorated Cognitive Decline in the APP/PS1 Mice

**Figure 1:**
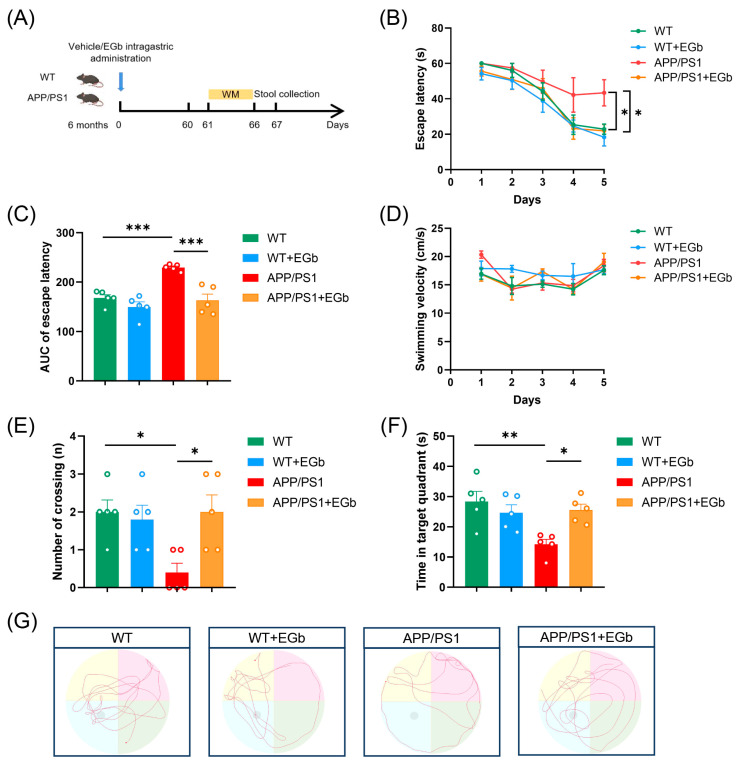
EGb ameliorates cognitive decline in the APP/PS1 mice. (**A**) Timeline of experimental procedure in our research. (**B**) Latency to escape to a hidden platform in the MWM task during the training phase. (**C**) The area under the curve (AUC) of escape latency during the training phase. (**D**) The swimming velocity of each group was recorded in the training phase. (**E**) The number of crossings into the border of the target zone in the probe trial. (**F**) The time spent in the quadrant containing the hidden platform in the probe trial. (**G**) Representative swimming trajectories of different groups of mice are shown. Data are presented as mean with SEM, *n* = 5, * p < 0.05, ** p < 0.01, *** p < 0.001 vs. APP/PS1 group. Data were analyzed with two-way ANOVA.

To determine whether EGb treatment affects behavioral deficits in AD, we administered EGb via the intragastric route to 6-month-old mice and analyzed their memory and spatial learning abilities through a Morris water maze task 2 months later (Figure 1A). During the training phase, EGb treatment reduced the escape latency, while the APP/PS1 group spent more time locating the hidden platform, indicating that EGb improved the learning ability of APP/PS1 mice (Figure 1B,C). All groups of mice had similar swimming velocities in the task (Figure 1D). In the probe trials, memory retention was measured as the time spent in the quadrant containing the hidden platform. In comparison to the APP/PS1 group, the EGb treatment group dramatically enhanced both the number of crossings over the target platform and the amount of time spent in the target quadrant (Figure 1E,F). Representative raw traces of swimming in the probe trial test are shown in Figure 1G. These results suggested that EGb improved spatial memory in the APP/PS1 mice.

### 3.2. EGb Reshaped Gut Flora Composition in APP/PS1 Mice

**Figure 2:**
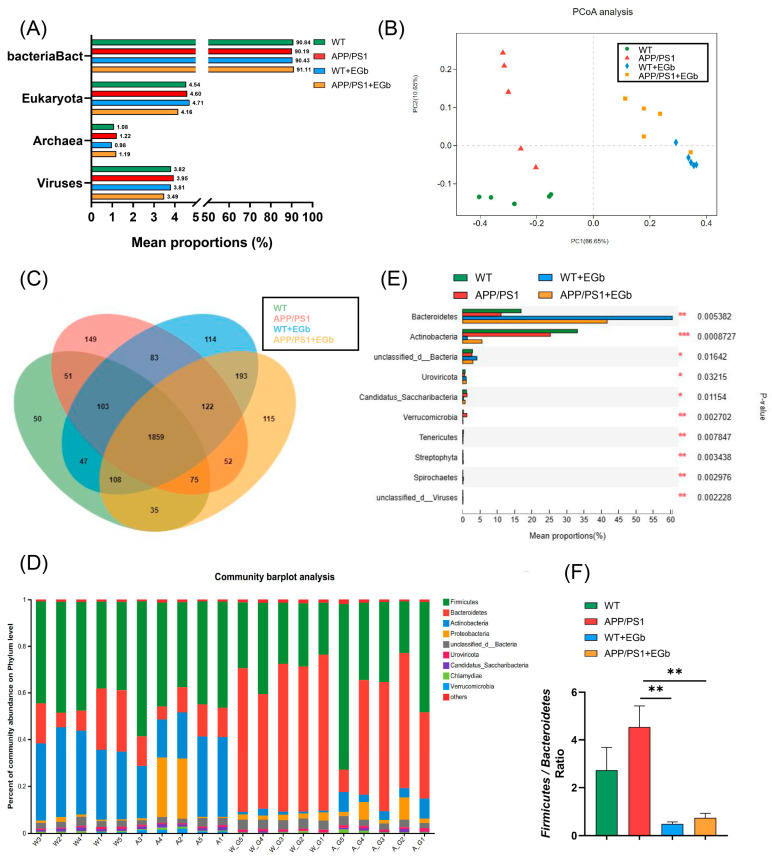
Gut flora composition alteration in WT and APP/PS1 mice treated with EGb. (**A**) Species annotation of the overall microbial. (**B**) Principal coordinate analysis (PCoA) of unweighted UniFrac revealed clustering of the gut flora. (**C**) A Venn diagram of the number of gut flora after treatment with EGb. (**D**) Stacked bar plots of the phylogenetic composition of bacterial taxa at the phylum level. (**E**) Difference analysis of the relative abundance at the bacterial phylum level. (**F**) *Firmicutes/Bacteroidetes* ratio at the phylum level. Data are presented as mean with SEM, *n* = 5, ** *p* < 0.01 vs. APP/PS1 group. Data were analyzed with two-way ANOVA.

The effects of EGb administration on the intestinal flora community structure of APP/PS1 mice were investigated by metagenomic sequencing analysis. Firstly, the species annotation results showed that the relative abundance of bacteria accounted for 89.83% of the total microbial relative abundance, while the values for eukaryotes, archaea, and viruses were 4.52%, 1.31%, and 4.29%, respectively, indicating that EGb does not change the overall microbial composition (Figure 2A). The results of the principal coordinate analysis (PCoA) at the genus level showed a distinct separation between drug-driven communities on the first principal coordinate, illustrating the overall microbiota community’s separation among the four groups (Figure 2B). A Venn diagram showed that 2108 OTUs (Operational Taxonomic Units) were shared between the APP/PS1 and APP/PS1 + EGb group, and there were 115 endemic bacteria after a 2-month EGb intervention (Figure 2C).

Species annotation abundance based on PCA was performed, and the community structure composition of all the bacteria in the four groups was analyzed at different taxonomic levels. A total of 192 phyla, 329 classes, 632 orders, 1147 families, 3232 genera, and 13,413 species were identified. Further, the histograms of the distribution and comparison analysis at the phylum level revealed enriched *Bacteroidetes, Uroviricota, Streptophyta,* and *Spirochaetes* abundances but decreased *Actinobacteria, Saccharibacteria, Verrucomicrobia,* and *Tenericutes* levels in the APP/PS1 + EGb group compared to the APP/PS1 group (Figure 2D,E). Numerous studies have identified the ratio of *Firmicutes* to *Bacteroidetes* as a maker of intestinal flora balance [25,26]. The relative abundance ratio of *Firmicutes*/*Bacteroidetes* was increased in the APP/PS1 group compared to the WT group, while EGb intervention decreased this (Figure 2F), suggesting that the progression of AD may be associated with a high proportion of *Firmicutes/Bacteroidetes* which could be lowered to normal by EGb treatment. These findings suggested that the microbial composition in the APP/PS1 group showed significant changes, while EGb could improve the abnormal microbial composition.

### 3.3. EGb Regulated the Specific Alterations in Gut Flora Diversity in APP/PS1 Mice

**Figure 3:**
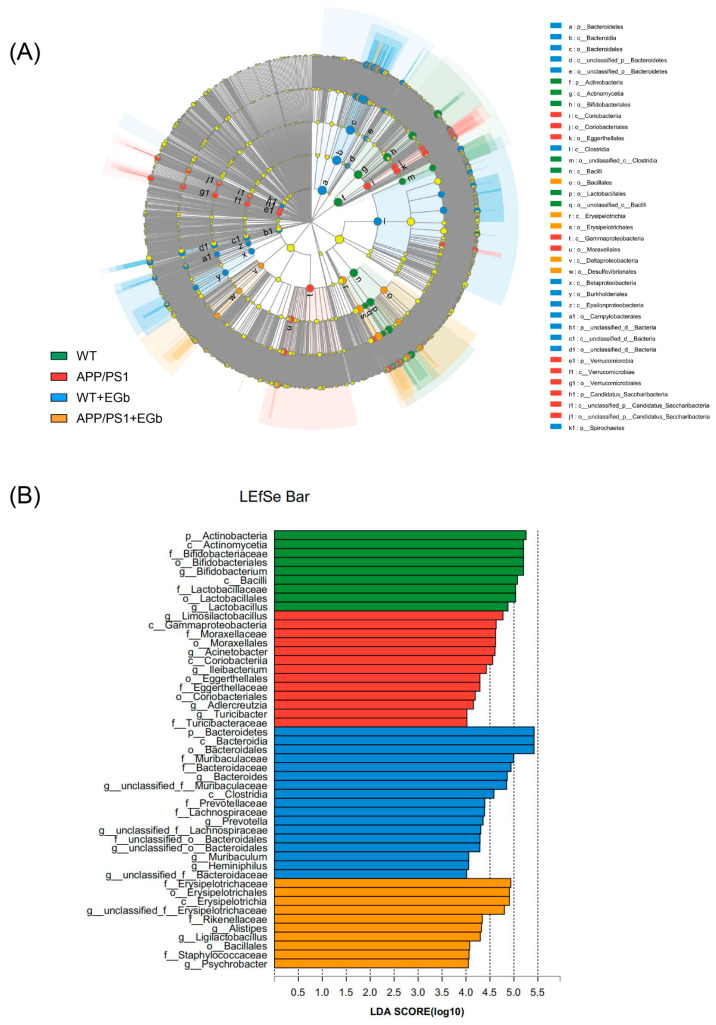
Discriminative taxa among the four groups. (**A**) The cladogram of the most differentially abundant taxa among the four groups. (**B**) The LDA scores of LEfSe analysis. The size of the point shows the negative logarithms (base 10) of the p-value. A larger point size shows greater significance (a lower *p*-value).

**Figure 4:**
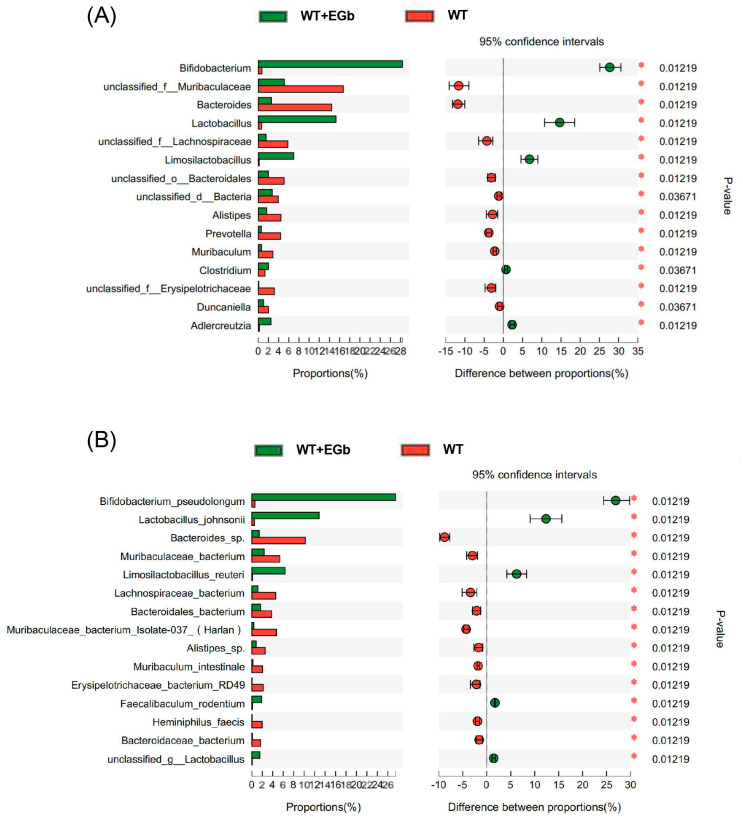
Specific alterations in gut flora diversity in WT mice treated with EGb. (**A**) Difference analysis of relative abundance at the bacterial genus level after EGb treatment in WT mice. (**B**) Difference analysis of relative abundance at the bacterial species level after EGb treatment in WT mice. *: *p* < 0.05.

The LEfSe multilevel species difference analysis of taxonomic alternation confirmed the dominant sets of microbes generated by EGb treatment, including 2 classes and 13 families, such as *Bacillales*, *Erysipelotrichia*, *Erysipelotrichales*, and *Deltaproteobacteria* (Figure 3A,B). The difference in bacteria flora at the genus level between the WT and WT + EGb groups was analyzed, which showed that *Bifidobacterium*, *Lactobacillus*, *limosilactobacillus*, *Clostridium*, and *Adlercreutzia* increased, while *Bacteroides*, *Alistipes*, *Alistipes*, *Muribaculum*, and *Duncaniella* decreased, after EGb treatment in the WT mice (Figure 4A). Additionally, EGb supplementation elevated the levels of *Bifidobacterium_pseudolongum, Lactobacillus_johnsonii*, *Limosilactobacillus_reuteri*, and *Faecalibaculum_rodentium* abundance at the species level and reduced the levels of *Bacteroides*_sp.*, Muribaculaceae_bacterium, Lachnospiraceae_bacterium*, *Bacteroidales_bacterium*, *Muribaculaceae_bacterium_Isolate-037_(Harlan), Alistipes*_sp., *Muribaculum_intestinale*, *Erysipelotrichaceae_bacterium_RD49*, and *Heminiphilus_faecis* abundance. Abundance describes the number of individuals of one or more species in a community of organisms (Figure 4B).

**Figure 5:**
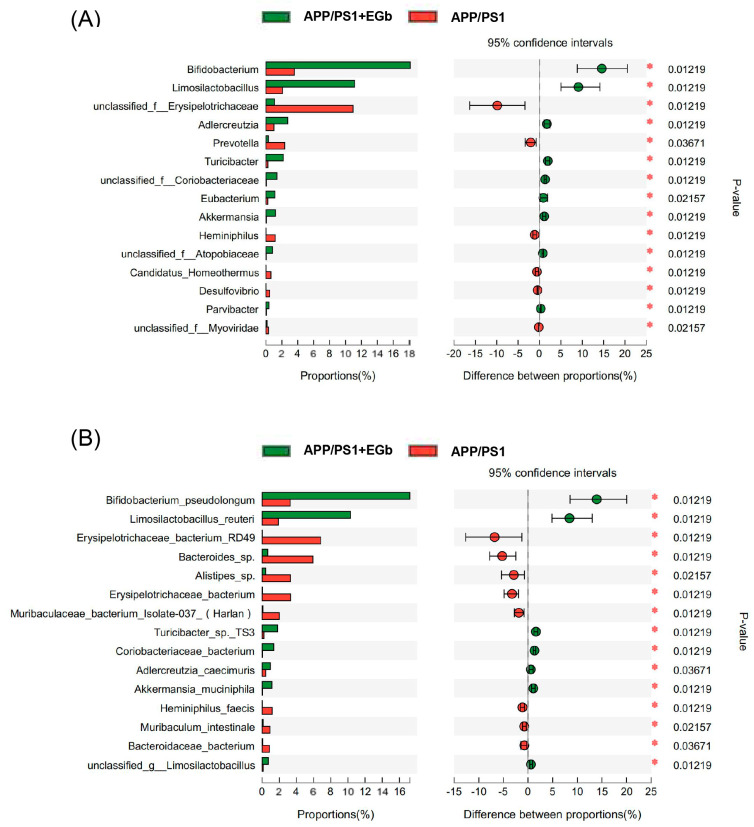
Specific alterations of gut flora diversity in APP/PS1 mice treated with EGb. (**A**) Difference analysis of relative abundance at the bacterial genus level after EGb treatment in APP/PS1 mice. (**B**) Difference analysis of relative abundance at the bacterial species level after EGb treatment in APP/PS1 mice. *: *p* < 0.05.

As shown in Figure 5A, the relative abundance of *Parvibacte*, *Heminiphilus*, *Candidatus_Homeothermus*, and *Desulfovibrio* in the EGb-treated group was less than that in the APP/PS1 group, whereas *Bifidobacterium*, *Limosilactobacillus*, *Adlercreutzia*, *Turicibacter*, *Akkermansia*, and *Parvibacte* were increased. Furthermore, the relative abundance of *Bifidobacterium_pseudolongum*, *Limosilactobacillus_reuteri*, *Turicibacter*_sp*._TS3*, *Coriobacteriaceae_bacterium*, *Adlercreutzia_caecimuris*, and *Akkermansia_muciniphila* increased in the EGb-treated group compared to the APP/PS1 group, whereas *Erysipelotrichaceae_bacterium_RD49*, *Alistipes*_sp., *Bacteroides*_sp., *Muribaculaceae_bacterium_Isolate-037_(Harlan)*, *Heminiphilus_faecis*, *Heminiphilus_faecis*, and *Bacteroidaceae_bacterium* decreased (Figure 5B). Together, EGb caused the most significant changes in *Bifidobacterium longum* and *Lactobacillus reuteri* in both the WT and APP/PS1.

### 3.4. EGb Treatment Altered Gut Metabolites in APP/PS1 Mice

**Figure 6:**
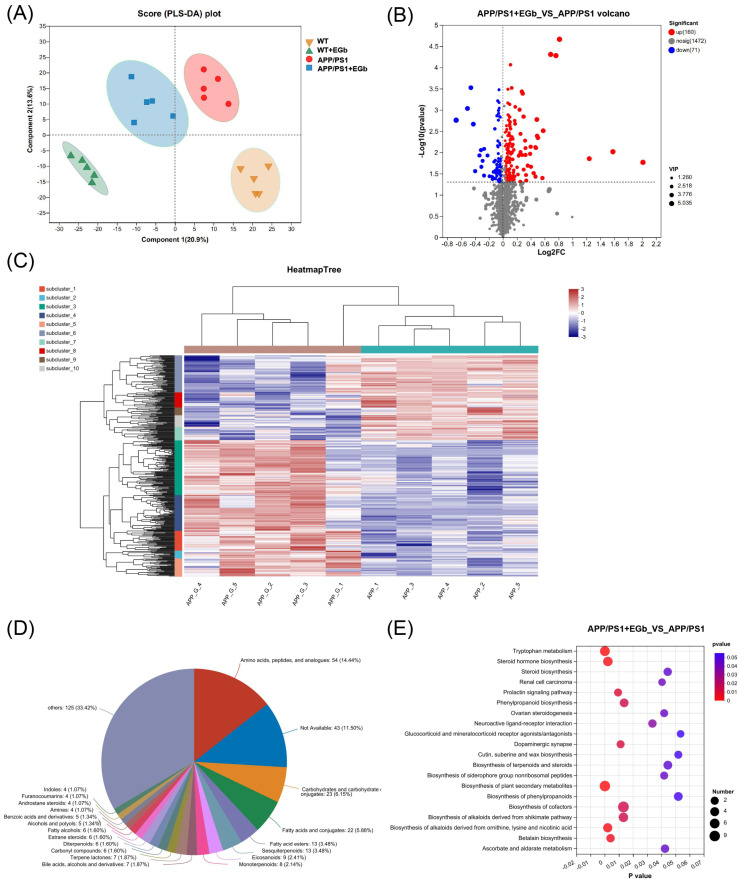
Intestinal metabolite alteration in APP/PS1 mice treated with EGb. (**A**) PLS-DA score plots of the metabolic profiles of WT and APP/PS1 mice with or without EGb. (**B**) Volcano diagram of the changes in metabolites in APP/PS1 mice after treatment with EGb. (**C**) The heat map generated by hierarchical clustering analysis for different comparison combinations with significant changes. (**D**) Global classification of intestinal metabolites in APP/PS1 mice treated with EGb. (**E**) KEGG enrichment analysis of the top 20 metabolic pathways in comparison combinations according to the *p*-value.

We carried out metabolomic analysis using UHPLC-QTOFMS and PLS-DA cluster analysis and determined the trends of metabolic changes after EGb treatment, relative to the WT and APP/PS1 groups. All samples were analyzed with a 95% confidence interval. The PLS-DA results showed that the four groups could be classified clearly (Figure 6A). We identified differentially expressed metabolites using significance analysis with VIP > 1 and *p* < 0.05, and the results were visualized in the form of volcano plots. There were 300 different metabolites in APP/PS1 vs. WT, including 131 increased and 169 decreased metabolites (Figure S1A, Table S1); 344 different metabolites in the EGb-treated WT vs. WT, including 191 increased and 153 decreased metabolites (Figure S1C, Table S2); and 231 different metabolites in the EGb-treated APP/PS1 vs. APP/PS1, including 160 increased and 71 decreased metabolites (Figure 6B, Table S3). In addition, the hierarchical cluster analysis and heat map of the differential metabolites in these two groups revealed that the subcluster relative abundance of the EGb-treated APP/PS1 mice showed a trend of half increase and half decrease (Figure 6C).

### 3.5. EGb Treatment Altered Gut Metabolic Pathways in APP/PS1 Mice

**Figure 7:**
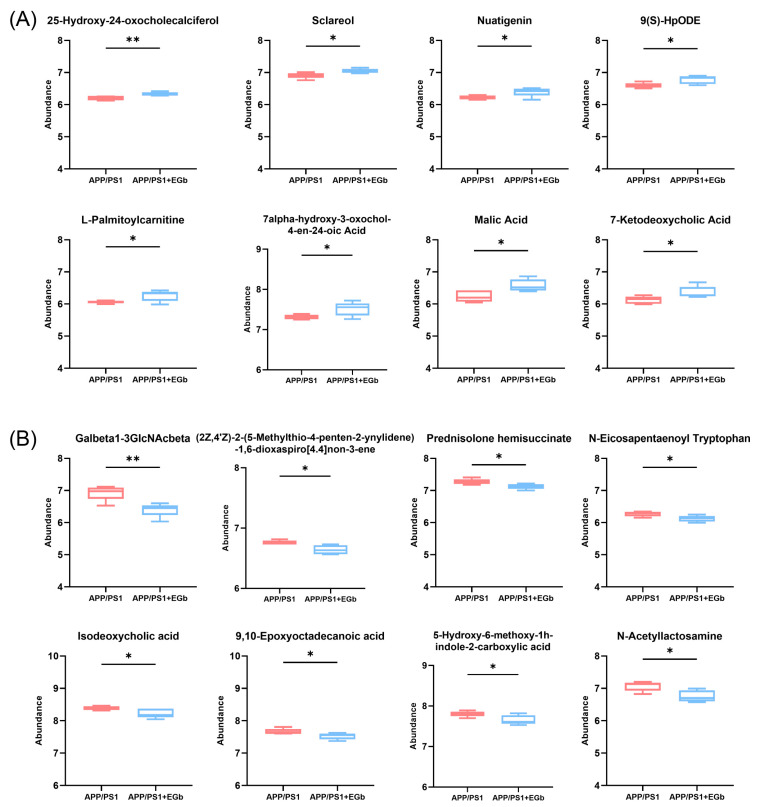
Comparison of intestinal metabolite relative abundance in APP/PS1 mice treated with EGb. (**A**) The top 8 metabolites with up-regulated relative abundance after EGb treatment compared with APP/PS1 group. (**B**) The top 8 metabolites with down-regulated relative abundance after EGb treatment compared with APP/PS1 group. Data are presented as mean with SEM, *n* = 5, * p < 0.05, ** p < 0.01. Data were analyzed with Student’s *t*-test.

A comparison with the Human Metabolome Database (HMDB) showed that, at the subcluster level (Figure 6D), the main differential metabolites were amino acids, peptides, and their analogs (14.44%); carbohydrates and carbohydrate conjugates (6.15%); fatty acids and conjugates (5.88%); fatty acid esters (3.48%); sesquiterpenoids (3.48%); eicosanoids (2.41%); monoterpenoids (2.14%); and terpene lactones (1.87%). Furthermore, the 300 differential metabolites in APP/PS1 vs. WT were enriched in 20 signaling pathways, mainly focused on α-Linolenic acid metabolism, steroid hormone biosynthesis, sphingolipid metabolism, and the serotonergic synapse (Figure S1B). The 344 differential metabolites in the EGb-treated WT vs. WT were enriched in 20 signaling pathways, focused on steroid hormone biosynthesis and the calcium signaling pathway (Figure S1D). The 231 differential metabolites in the EGb-treated APP/PS1 vs. APP/PS1 were enriched in 20 signaling pathways, focused on tryptophan metabolism, steroid hormone biosynthesis, neuroactive ligand–receptor interaction, the dopaminergic synapse, and the biosynthesis of terpenoids and steroids (Figure 6E). Furthermore, compared with the APP/PS1 group, the top eight metabolites with an up-regulated relative abundance after EGb treatment are shown in Figure 7A, including 25-Hydroxy-24-oxocholecalciferol, Sclareol, Nuatigenin, 9(S)-HpODE, L-Palmitoylcarnitine, 7α-hydroxy-3-oxochol-4-en-24-oic acid, Malic acid, and 7-Ketodeoxycholic Acid. The top eight metabolites with a down-regulated relative abundance after EGb treatment are shown in Figure 7B, including Galbeta1-3GlcNAcbeta, Prednisolone hemisuccinate, N-Eicosapentaenoyl tryptophan, (2Z,4′Z)-2-(5-Methylthio-4-penten-2-ynylidene)-1,6-dioxaspiro[4.4]non-3-ene, Isodeoxycholic acid, 9,10-Epoxyoctadecanoic acid, 5-Hydroxy-6-methoxy-1h-indole-2-carboxylic acid, and N-Acetyllactosamine.

### 3.6. Association Analysis of Metagenomic and Metabolomic Profiles

**Figure 8:**
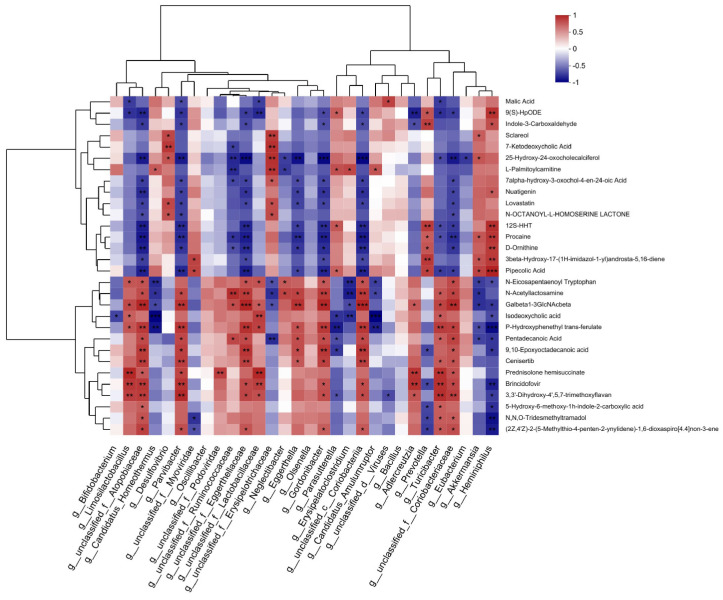
Correlation between gut microbiota and metabolites. Correlation analysis at the genus level of the 30 most abundant gut microbiota and 30 different metabolites. * p < 0.05, ** p < 0.01, *** p < 0.001. Positive correlation is indicated in red and negative correlation is in blue; *n* = 5.

To further investigate the relationship between AD metagenomes and multiple metabolites, we analyzed the gut microbiota and metabolites in APP/PS1 mice. Firstly, the intestinal microorganisms of different species in the EGb-treated and saline-treated APP/PS1 groups were screened, and then a correlation analysis was conducted with the different metabolites in the two groups. A correlation analysis of the top 30 gut microbiota and 30 different metabolites is presented in Figure 8, Table S4. The results showed that three gut microbiota (*g_Oscillibacte*, *g_Olsenella*, *g_Bacillus*) were not associated with any metabolite, and few were associated with a single metabolite, including *g_Bifidobacterium* and *g_Eubacterium*, which had a negative correlation with Isodeoxycholic acid and 25-Hydroxy-24-oxocholecalciferol, respectively, while *g_unclassified_f_Podoviridae* showed a positive correlation with Prednisolone hemisuccinate. Others were associated with a variety of metabolites, suggesting that different species of intestinal flora may affect different intestinal metabolic processes and ultimately influence the pathophysiology of AD mice.

In order to further study the intrinsic relationship between the differential metabolites and intestinal microbiota, significantly correlated metabolites and microorganisms were extracted from each group. Combined with related metabolic pathway analysis, an interaction network diagram of the metabolite–microbiome pathway was constructed. The results showed that EGb treatment was mainly related to lipid metabolism, chemical structure transformation maps, and the biosynthesis of other secondary metabolites. The intestinal microbiota, mainly five kinds of bacteria, including *g_Turicibacter*, *g_Limosilactobacillus*, *g_unclassified_f_Lactobacillaceae*, *g_unclassified_f_Eggerthellaceae*, and *g_Parvibacter*, were mostly involved in the above metabolic pathways.

**Figure 9:**
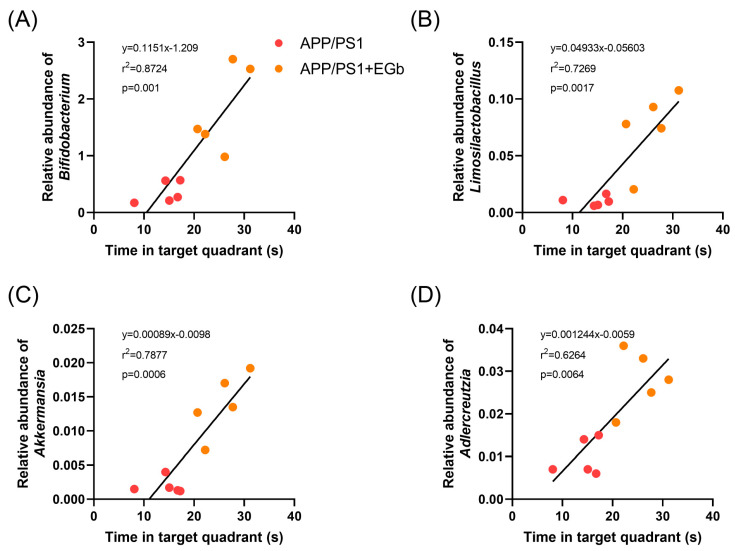
Correlation between gut microbiota and behavior results. Correlation analysis between (**A**) *Bifidobacterium*, (**B**) *Limosilactobacillus*, (**C**) *Adlercreutzia*, and (**D**) *Akkermansia* and time in target quadrant of APP/PS1 mice before and after EGb treatment.

We analyzed the correlation between the behavior results and significantly improved probiotics before and after EGb treatment. The results showed that all four kinds of probiotics, *Bifidobacterium*, *Limosilactobacillus*, *Adlercreutzia*, and *Akkermansia*, were positively correlated with the results of the water maze test, suggesting that the relative abundance of intestinal probiotics in APP/PS1 mice was significantly increased after EGb treatment, which further affected their learning and memory behavior, and there was a correlation between intestinal probiotics and cognitive dysfunction (Figure 9). In addition, fecal bacteria transplantation experiments have confirmed that probiotics can improve the cognitive function of AD model mice [27]. In summary, these results indicated that the EGb treatment affected the relative abundance of several intestinal flora and regulated the levels of related metabolites, thereby improving the pathology of AD.

## 4. Discussion

Numerous studies have shown that changes in the gut microbiome are associated with AD, as they involve a wide range of functions, including the regulation of neuroinflammation [28] and impacts on metabolic functions [29], as well as immune responses [30]. Currently, targeting of the intestinal microbiota has been proposed as a prospective therapeutic strategy to ameliorate the pathology of AD [31]. *Ginkgo biloba* extract (EGb) has been shown to alleviate neurodegenerative diseases, depression [32], and atherosclerosis [33] and restore intestinal integrity through the brain–gut axis [34]. However, its precise mechanism of action has not yet been elucidated. In our research, for the first time, we demonstrated the amelioration of memory deficits and revealed changes in, and the relationship between, the gut microbiome and metabolites in APP/PS1 mice after EGb treatment.

Metagenomic sequencing was performed to detect the composition and diversity of intestinal microflora in the WT and APP/PS1 mice after EGb treatment. EGb could establish a balanced intestinal flora. In particular, supplementation with EGb increased the relative abundance of *Bifidobacterium_pseudolongum*, *Limosilactobacillus_reuteri*, *Turicibacter*_sp._*TS3*, *Coriobacteriaceae_bacterium*, *Adlercreutzia_caecimuris*, and *Akkermansia_muciniphila,* which have recently been reported to have health-related effects. For instance, *Bifidobacterium_pseudolongum* could reduce the level of pro-inflammatory factors and maintain the integrity of the intestinal physical barrier [35,36]. Additionally, it can regulate the immune system, alter the local or systemic immunological response in a strain-specific manner, and trigger immune cell secretion [37,38,39]. A beneficial probiotic microbe called *Limosilactobacillus_reuteri* modulates the intestinal milieu by producing antimicrobial compounds, enhances insulin sensitivity and glucose balance, and alleviates metabolic disorders [40,41]. It can repair intestinal epithelial damage and protect the integrity of the intestinal mucosal barrier. Moreover, individuals with early cognitive dysfunction develop brain capillary damage and blood–brain barrier (BBB) breakdown in the hippocampus, while BBB damage results in a breakdown of brain homeostasis that leads to neuroinflammation, neuronal dysfunction, and, ultimately, severe neurological diseases, and supplementation with maternal *Limosilactobacillus_reuteri* from birth improves neurodevelopmental impairments and recovers cognitive performance linked to BBB dysplasia and malfunction [42]. In addition, studies have shown that the probiotic *Lactobacillus* could control purine metabolism in the gut, relieve colitis, and enhance depressive and anxiety-like symptoms, hence alleviating mental illness [43,44]. Our results suggest that EGb improves cognitive impairment following long-term dosing by stimulating the growth of advantageous bacteria and controlling intestinal homeostasis.

Microbial metabolites are key mediators of microbe–host crosstalk, significantly affecting the metabolism of organisms. The intestinal microbiota produces metabolites such as tryptophan, short-chain fatty acids (SCFAs), lipids, and other secondary metabolites, which are involved in the regulation of host metabolism and gut integrity [45,46,47]. Currently, many studies are focusing on the effects of SCFAs on AD, and this study focused on the effects of tryptophan, steroids, and other metabolites on AD pathology. In this study, all the metabolites in APP/PS1 mice treated with or without EGb were examined. Our results demonstrate that the host has enriched tryptophan metabolism, steroid hormone biosynthesis, and neuroactive ligand–receptor interactions. According to a previous study, the levels of tryptophan metabolites, inflammatory cytokine release, and oxidative damage in the brain of triple-transgenic (3xTg-AD) mice increased [48]. Tryptophan metabolism is mediated by the bacteria *Bifidobacterium longum* and *Lactobacillus reuteri*, which inhibit Aβ deposit and tau hyperphosphorylation, reduce inflammatory cytokine levels, and consequently restore synaptic plasticity [49]. Neurosteroids gradually decrease with age due to the accumulation of Aβ precursor protein (APP) in the brain. Significant changes in steroid hormone production have been observed in AD patients [50,51], as have down-regulated steroid hormones in the stool of APP/PS1 mice. Additionally, a previous study showed that medications enhance memory impairment by connecting anti-apoptotic pathways and altering the expression of genes linked to neuroactive ligand–receptor interactions [52]. The results of our study prove that EGb can influence the aforementioned metabolic processes. We also examined all metabolites in WT mice with or without EGb. Our findings suggest that the host is enriched in steroid hormone biosynthesis and the calcium signaling pathway. Steroid hormones help to control metabolism, many steroid hormones have a good function of regulating sugar metabolism as well as water and salt metabolism, and a variety of steroid hormones have excellent anti-inflammatory and anti-allergic properties [53].

EGb contains 24% flavonoids and 6% terpenoid lactone, and the active components in the extract have also shown anti-neuroinflammation functions and capacities for protection against vascular damage and the amelioration of cognitive impairment in a variety of in vivo and in vitro models. Among them, flavonoids such as apigenin, quercetin, rutin, kaempferol, gallic acid, etc., can down-regulate the level of inflammatory factors in the brain, relieve neuroinflammation, and improve spatial memory disorders [54]. Lactones can enhance cerebral blood flow, boost the number of cerebral microvessels, and prevent cerebrovascular damage [55,56]. Thus, further research is required to determine the precise neuroprotective effects of active components of EGb in the “brain–gut” axis.

The main highlight of this study is that, for the first time, we used metagenomic and metabolomic analyses to show that EGb treatment can increase gut probiotic production and boost the levels of related metabolites in AD mice. Our study also demonstrates the importance of metabolites other than SCFA, such as neurosteroids, in the treatment of AD. Our study has certain possible limitations, and our goal in future research will be to verify the mechanism of action of the pertinent flora and metabolites. We expect to offer the fundamental knowledge required for the therapeutic application of EGb in the prevention and treatment of AD.

## 5. Conclusions

EGb ameliorated cognitive decline in APP/PS1 mice after 10 weeks of intragastric administration, remodeled the intestinal flora of the mice, significantly increased the abundance of *Bifidobacterium_pseudolongum* and *Limosilactobacillus_reuteri*, and regulated the specific changes in intestinal flora diversity. In addition, it regulates tryptophan metabolism, steroid hormone biosynthesis, and neuroactive ligand–receptor interactions. In conclusion, EGb improves the pathology of AD by increasing the production of intestinal probiotics and promoting the levels of related metabolites in AD mice.

## Data Availability

All data are available from the corresponding authors upon reasonable request.

## Supplementary Materials

The following supporting information can be downloaded at: https://www.mdpi.com/article/10.3390/pharmaceutics15122746/s1, Figure S1: Intestinal metabolite and metabolic pathways alteration. A, Volcano diagram of the changes in metabolites between APP/PS1 and WT mice. B, KEGG enrichment analysis of the top 20 metabolic pathways in comparison combinations according to the P value. C, Volcano diagram of the changes in metabolites between WT+EGb and WT mice. D, KEGG enrichment analysis of the top 20 metabolic pathways in comparison combinations according to the *p* value. Table S1. 300 different metabolites between WT and APPPS1 mice. Table S2. 344 different metabolites between WT+EGb and WT mice. Table S3. 231 different metabolites between APPPS1+EGb and APPPS1 mice. Table S4. Correlation between gut microbiotas and metabolites.
