# Supplementary material for: Ginkgo biloba Extract Drives Gut Flora and Microbial Metabolism Variation in a Mouse Model of Alzheimer’s Disease
Source: Pharmaceutics. 2023 Dec 8;15(12):2746. doi: 10.3390/pharmaceutics15122746 (PMC10747232; doi:10.3390/pharmaceutics15122746)
Supplement: Supplementary file 1 [file pharmaceutics-15-02746-s001.zip › pharmaceutics-2758753-supplementary.pdf]

# *Ginkgo biloba* Extract drives gut flora and microbial metabolism variation in a mouse model of Alzheimer’s disease

## *Supplemental Information*

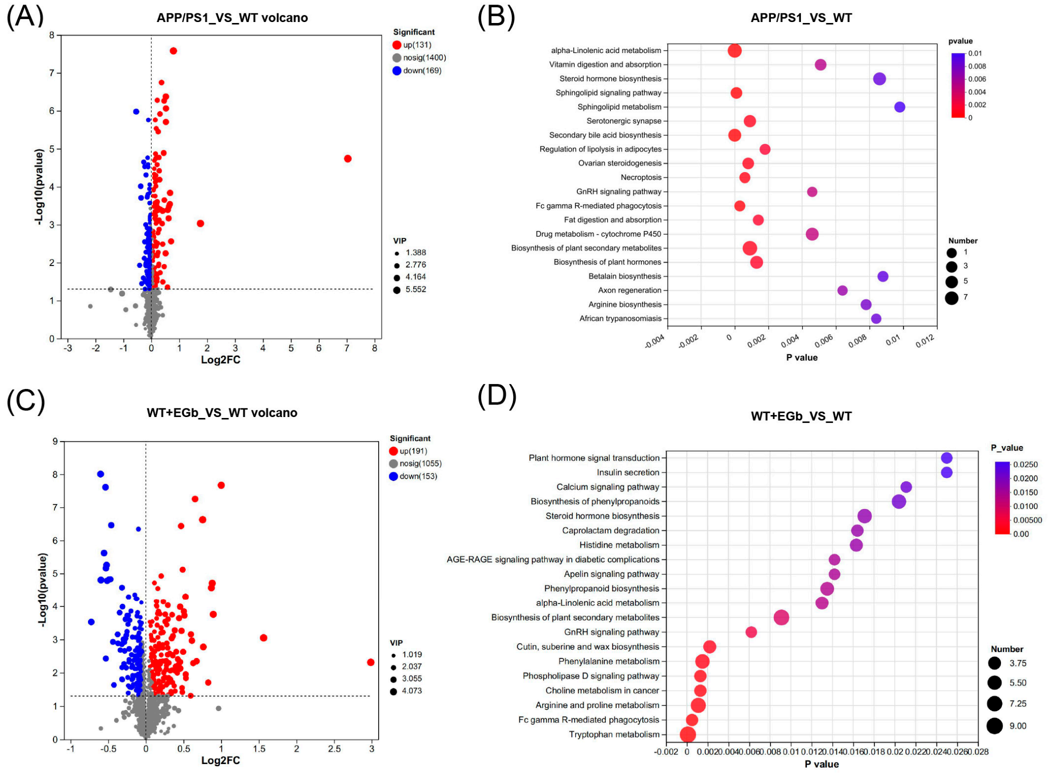


**Figure S1** Intestinal metabolite and metabolic pathways alteration. A, Volcano diagram of the changes in metabolites between APP/PS1 and WT mice. B, KEGG enrichment analysis of the top 20 metabolic pathways in comparison combinations according to the *P* value. C, Volcano diagram of the changes in metabolites between WT+EGb and WT mice. D, KEGG enrichment analysis of the top 20 metabolic pathways in comparison combinations according to the *P* value.
